# Incidence of trapezius myofascial trigger points in patients with the possible carpal tunnel syndrome

**Published:** 2010

**Authors:** Hamid Azadeh, Mohammad Dehghani, Abolghasem Zarezadeh

**Affiliations:** a Assistant Professor of Clinical Neurophysiology, Physiotherapy Department, School of Rehabilitation Science, Isfahan University of Medical Sciences, Isfahan, Iran; b Associate Professor of Orthopedic Surgery, Department of Orthopedics, School of Medicine, Isfahan University of Medical Sciences, Isfahan, Iran; c Assistant Professor of Orthopedics, Department of Orthopedics, School of Medicine, Isfahan University of Medical Sciences, Isfahan, Iran

**Keywords:** Carpal Tunnel Syndromes, Myofascial Trigger Point, Electrodiagnoses

## Abstract

**BACKGROUND:**

Patients with carpal tunnel syndrome (CTS) often complain of prominent pain in shoulder and arm, also there are patients that have pain in their shoulder and arm which is due to myofascial trigger point (MTP) located in their upper trapezius muscle. Despite the frequency of this observation, few studies have previously sought to establish possible relationship between the CTS and MTP in shoulder area.

**METHODS:**

Samples were 160 patients (221 hands) consist of 130 females and 30 males, with suspected diagnosis of CTS, from March 2008 to October 2008. In this study after performing electrodiagnosis searches, another evaluation was performed to find out if there was any sign of myofascial trigger point. The correlation between these two was sought.

**RESULTS:**

It was found that all of 36 hands with normal electrodiagnostic findings had myofascial trigger points in their upper trapezius muscle. Out of 185 hands, 130 hands (70%) with electrophysiological evidences of CTS showed myofascial trigger points in their trapezius muscles. Statistical analysis revealed significant (p < 0.001) reverse correlation between the severity of CTS and the presence of MTP.

**CONCLUSIONS:**

The findings of this study imply the significant correlation between occurrence of CTS and MTP. It is suggested that clinicians consider the probability of existence of MTP in patients referred for diagnosis of CTS.

Carpal tunnel syndrome (CTS), the most common focal peripheral neuropathy, results from compression of the median nerve at the wrist.^1^–^7^

Many patients referred to an electrodiagnostic laboratory for evaluation of possible CTS might have other complaints, most commonly musculoskeletal disorders.^8^–^10^

The spots are tender in palpation and can produce referred pain, and tenderness, motor dysfunction and autonomic phenomena.^11^–^15^

Active and latent are two subdivisions of MTP.^16^,^17^

Acute trauma or repetitive microtrauma may lead to the development of a MTP.^12^,^18^

Some studies have mentioned the abnormal electrodiagnosis findings and presence of MTP in different muscles.^19^–^21^

Other studies have reported the presence of active and latent MTP in trapezius muscles and the effect of injection in these points.^22^,^23^ This study was conducted to determine the proportions of negative and positive electrodiagnostic findings in patients referred to electrodiagnostic center for evaluation of clinically suspected CTS. Also it was meant to determine the incidence of MTP in CTS and any correlation between the severities of CTS based on clinical and electrophysiological criterion and presence of MTP.

## Methods

After institutional approval and receiving patients’ written consent, 160 adult patients were selected for study. They referred to electrodiagnostic center for evaluation of possible CTS, from March 2008 to October 2008. These patients were referred from orthopedic surgeons, neurologist, neurosurgeons and rheumatologist. Patients’ signs and symptoms and occupation were collected from the referrals and from the patients’ reports at the time of study. Information about paresthesia and pain and presence of hypoesthesia in hands and shoulder were obtained during the examination. Those patients who had pain due to fibromyalgia, tendinitis around shoulder girdle, herniated disc as well as degenerative joint disease at cervical spine, rheumatoid arthritis or pancoast syndrome were excluded. The study was approved by Ethics Committee of Isfahan University of Medical Sciences.

Neurophysiological evaluations were performed by the same investigator using an Easoate Biomedics 4 channels electromyograph (Phaysis II Myoto, Italia 1997). NCS were done by surface electrodes and included (a) antidromic sensory studies (conduction velocity and amplitude) of median and ulnar nerve from wrist to respective fingers and (b) motor studies (distal latency, amplitude and conduction velocity) of median nerve from the wrist to abductor pollicis brevis muscles and of ulnar nerve from the wrist and the forearm to abductor digiti minimi muscle. All standard tests for CTS were performed according to American Association of Electrodiagnostic Medicine (AAEM) guidelines.^24^ To exclude proximal lesions of the median nerve, thoracic outlet syndrome, radiculopathy and polyneuropathy, nerve conduction studies of the ulnar and radial nerves and EMG of forearm and paraspinal muscles was also conducted.

All hands tested were divided into six classes of severity based on the neurophysiological abnormalities:

0- “Negative” (NEG): normal findings in all tests including comparison to the ulnar nerve and short segmental tests.
1- “Minimal CTS” (MIN): abnormalities only when compared to the ulnar nerve or tested by short segmental studies
2- “Mild CTS” (MILD): slowing of sensory conduction across digit-wrist segment but normal distal motor latency (DML)
3- “Moderate CTS” (MOD): slowing of sensory conduction across digit-wrist segment and increased DML.
4- “Severe CTS” (SEV): absence of median SNAPs and increased DML.
5- “Extremely severe CTS” (EXT): absence of thenar motor and sensory response.

All patients with CTS underwent clinical assessment for MTP.

Patients were examined for MTP by a blinded examiner just after NCS was done.

According to Simons et al,^11^ the diagnostic criteria for the presence of myofascial trigger point were as follow: taut band, tender nodule on the taut band with increased pain on pressure, pain recognition, referred pain, and local twitch.

### 

#### Statistics

Statistical analysis included independent t test and non parametric test such as Chi Square, Mann-Whitney and ANOVA tests were used for non-normal distributed data. P value < 0.05 was considered to be significant.

## Results

**Figure 1:**
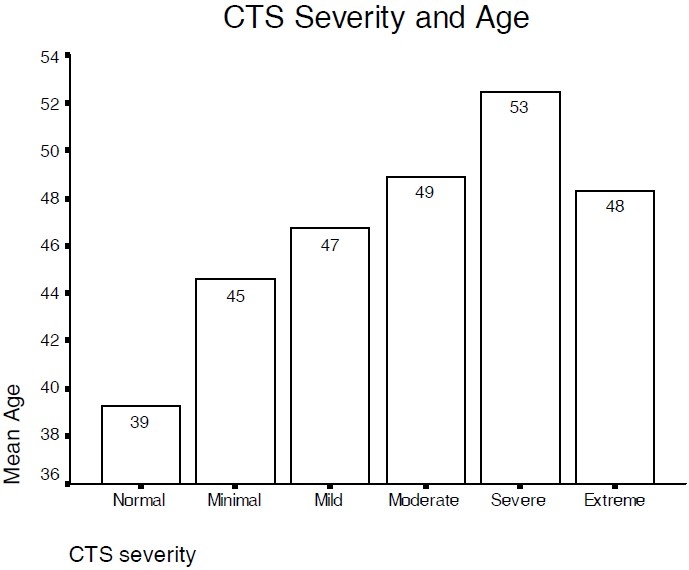
Mean ages in patients with different severity of CTS

A total of 221 hands (122 right and 99 left hands) from 160 patients, 130 females and 30 males, with mean age of 46 ± 13 years old (Range: 16-75) were included in the study. Of 221 hands with clinical diagnosis of CTS, 36 hands (16.3%) had no electrophysiological abnormality exhibited MTP in their Trapezius muscles. Of the remaining 185 hands with electrophysiological evidences of CTS, 130 showed MTP (70%) and 55 (30%) did not have MTP. In general, as the severity of CTS increased, the incidence of MTP decreased. Of 156 hands with minimal to moderate CTS, 121 (77.6%) presented MTP whereas out of 29 (69%) with severe and extreme CTS, only 9 (31%) showed evidence of MTP. Spearman test revealed significant (p < 0.001) reverse correlation between the severity of CTS and the presence of MTP (Table 1) (Figure 1).

**Table 1:** MTP in different CTS Severity classes

	CTS severity			Presence of MTP		
	Severity	Frequency	Percent	No MTP	With MTP
0	Normal	36	16.3%	0 (0%)	36 (21.7%)
1	Minimum	64	29.0%	12 (21.8%)	52 (31.3%)
2	Mild	50	22.6%	7 (12.7%)	43 (25.9%)
3	Moderate	42	19.0%	16 (29.1%)	26 (15.7%)
4	Severe	26	11.8%	19 (34.5%)	7 (4.2%)
5	Extreme severe	3	1.4%	1 (1.2%)	2 (1.8%)

	Total	221	100%	55 (100.0%)	166 (100.0%)

**Figure 2:**
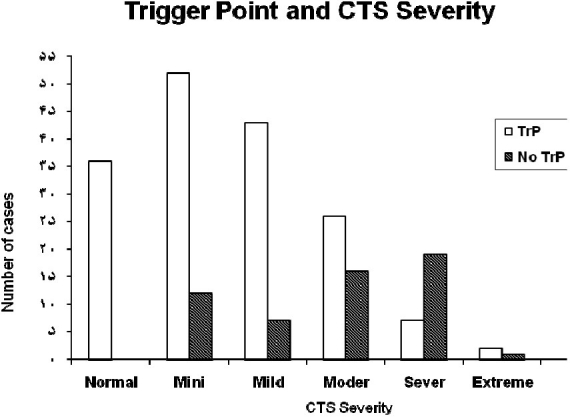
MTP in different severity of CTS are present

Also a possible relationship between the severity of CTS and age was assessed. The mean age was significantly (p < 0.005) greater in more advanced CTS groups “Severe and Extremely severe” as compared to less advanced CTS cases (Moderate, Mild and Minimal groups)(Figure 2).

**Figure 3:**
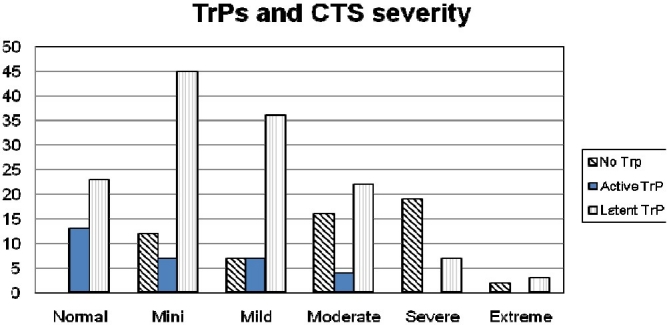
Number of patients who had MTP with different severity of CTS

As the patients became older, the presence of MTP was less and conversely younger patients showed a higher prevalence of MTP (p < 0.001). There were 55 limbs (24.9%) without MTP, and 166 limbs (75.1%) with MTP in the area of the shoulder. From all upper limbs with MTP, 31 (22.8%) had active and 135 (77.2%) had latent MTP (Figure 3).

**Figure 4:**
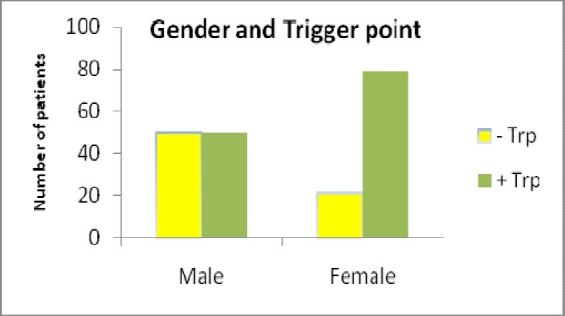
The incidence of MTP in both genders

Statistical analyses showed a reverse correlation between presence of MTP and patients’ ages. Presence of MTP was 50% in males and was %79.1 in females (Figure 4).

## Discussion

This single-blinded study searched the occurrence of myofascial trigger point in the upper trapezius muscle in a patients population (166 patients with 221 hand involved) referred for CTS. Thirty six hands (21.7%) showed normal electrophysiological findings but all had MTP in the upper trapezius muscle associated with paresthesia and pain referred to the arm and fingers. In these cases MTP possibly would explain their symptoms. This study is in line with Lo et al study^20^ as well as Qerama et al.^21^ The current study confirm the results of Padua et al study,^25^,^26^ who emphasized the importance of differentiating CTS from musculoskeletal disorders by performing a comprehensive musculoskeletal history and physical examinations. The results of the present study suggest that a patient with CTS may also have MTP as Qerama noticed it too.^21^ Also the present results are in favor of Davies findings^19^ but in this study the trapezius muscles showed the place where MTP are located and not scalenous or Infraspinatus muscles.^21^ All of the present patients with normal NCV (36 cases) showed MTP, which is twice the percentage that Qerama noted.^21^ They did not mention that they have included latent MTPs or just active ones. Infraspinatus muscle is a layer beneath the trapezius and latissimus dorsi muscles, so finding the exact location of MTP might be confusing which is in favor of literature review that Lucas et al performed.^27^ Presence of MTP in patients with different intensity of CTS was found here, which is not consistent with the results of Lo et al^20^ ; they postulated that patient who do not have CTS, might have musculoskeletal involvement, and they did not search the presence of MTP in their CTS subjects. MTPs in CTS patients should be considered as a secondary phenomenon and not a different pathology associated with it.

## Conclusions

In conclusion, electrodiagnostic evaluations play an important role in the assessment of CTS. Patients with CTS undocumented by neurophysiologic studies are often prone to further diagnostic tests such as cervical spine CT and/or MRI. Before ordering the expensive studies, it is best to search for MTP, which may respond to appropriate therapy. Therefore, further studies are suggested to explore the possible relationship between CTS and MTP, reverse correlation between CTS severity and MTP and the affect of patients’ ages with these two disorders.
